# Using Mediterranean Native Plants for the Phytoremediation of Mining Sites: An Overview of the Past and Present, and Perspectives for the Future

**DOI:** 10.3390/plants12223823

**Published:** 2023-11-10

**Authors:** Maria Enrica Boi, Mauro Fois, Lina Podda, Marco Porceddu, Gianluigi Bacchetta

**Affiliations:** Sardinian Germplasm Bank (BG-SAR), Centre for the Conservation of Biodiversity (CCB), Department of Life and Environmental Sciences, University of Cagliari, 09123 Cagliari, Italy; menricaboi@gmail.com (M.E.B.); lina.podda@unica.it (L.P.); porceddu.marco@unica.it (M.P.); bacchet@unica.it (G.B.)

**Keywords:** metal(loid) pollution, Mediterranean vascular flora, metallophytes, environmental restoration, mine wastes

## Abstract

Mining exploitation in the Mediterranean Basin has left evident scars on the environment, and poses serious risks for human health and biodiversity, especially when mine wastes are left abandoned. This review analysed the main issues of metal(loid)s pollution related to mine exploitation in the Mediterranean Basin. Here, a list of Mediterranean native plant species studied for phytoremediation is given and, considering their biological forms, vegetational types, and ecology, we categorised them into halotolerant and hydro/hygrophilous vegetation, annual and perennial meadows, garrigues and maquis, and high maquis and woods. The main conclusions of the review are as follows: (1) plant communities established on mine environments are often rich in endemic *taxa* which ensure a high biodiversity and landscape value, and can help in the psychophysical health of local inhabitants; (2) political and land management should take greater account of the use of native plants for the remediation of contaminated soils; (3) a multidisciplinary approach that includes, among others, studies on biochemical response to metal(loid)s as well as the application of innovative soil amendments gives better results; (4) phytoextraction applications require a detailed recovery plan that takes into consideration several issues, including the negative influence on biodiversity due to extensive use of monotypic plantations, disposal of harvested hazardous plants, and the risk of phytoextracts entering the food chain; and (5) more studies are necessary to increase knowledge and to detect suitable species—especially halophytic ones—for phytoremediation purposes.

## 1. Introduction

The environmental impact of mining activities, both historical and ongoing, has left important traces on the landscape across the world. Evidence of this can be seen in the form of waste mine dumps, pits, and quarries that persist as enduring features in many regions. The Mediterranean Basin has not been spared from these detrimental effects [1,2,3,4]. The Mediterranean biogeographical region is considered a global biodiversity mega-hotspot, being particularly rich in endemic plants, which are often threatened by human pressures and climate change [5]. It is also considered as one of the regions most devoted to mining exploitation, since pre-Roman times, for the extraction of Ag, Au, Cu, Fe, Pb, and Zn, and other minerals, like bauxite, galena, and pyrite [6,7]. Nevertheless, the extensive exploitation of the historically richest ores started only with the industrial revolution in the XIX century. These mines are now largely abandoned all over the Mediterranean Basin, even if some are still active (e.g., on the Troodos Mountains in Cyprus or in different sites of the Maghreb, NW Africa). The waste materials were often left to weathering without reclamation, leaving lasting scars on the environment, and impacting on the landscape, biodiversity, and the well-being of local communities. These consequences have far-reaching implications for both the present and the future, necessitating a comprehensive understanding of the challenges at hand and the potential solutions [8,9].

Considering that mine sites are often in close proximity to rural villages and urban areas, policy guidelines and land management must be carefully designed in order to safeguard the health of inhabitants as a priority issue, and to develop suitable conditions for improving the well-being and quality of life, also in accordance with the most recent European green deal, the EU Biodiversity Strategy for 2030 [10], and the European guidelines on biodiversity-friendly afforestation, reforestation, and tree planting [11]. Hence, in the frame of the green-circular economy, the reclamation of mine sites through phytoremediation is nowadays well supported by the scientific community. In the last twenty years, several important efforts have been made in the Mediterranean region [12]. Many of these consider only one aspect of the issue, although a multidisciplinary approach, involving geochemistry, biochemistry, geobotany, environmental engineer, mineralogy, and microbiology together, is needed in order to better shed light on the complex interactions between plants and soil/waste materials. For instance, even if many plants can accumulate highly toxic metal(loid)s, only hyperaccumulators can absorb high concentration of them, and transport them from roots to xylem and then to frond cells. Nonetheless, the phytoavailability of metal(loid)s depends on many factors, such as soil chemistry, texture, and the availability of plant-associated microbial communities, which contribute to the tolerance, sequestration, and transport of metal(loid)s by plants [12,13,14]. The choice of the most suitable *taxa* is of crucial importance for effective action. In this frame, the use of native plants is preferable for their possible high adaptability and to preserve the natural plant assemblage of territories [15,16,17,18]. Consequently, knowledge about the vegetation dynamics of mining environments can provide essential information for the planning of efficient and effective remediation plans [19].

Basic research towards the description and definition of further metallophytes has been more recently complemented by the individuation of technologies to assist and boost phytoremediation, such as the development of substrate amendments or modifiers. In particular, attention has been oriented to the use of innovative amendments, often deriving from the re-use of other waste materials (i.e., waste from agricultural production, wood chips, biochar, and mushroom residues) [20,21,22,23,24,25]. The combination of modifiers is another branch of study showing promising results [20,26,27]. A great contribution is also offered by microbiology with bioaugmentation technologies, with selected bacterial strains native on the roots of the investigated plant or of the local substrate [14,28], as well as the implementation of synchrotron-based techniques for the development of molecular environmental science (MES), that allows researchers to understand, at the molecular scale, the phenomena controlling environmentally relevant processes such as the formation of biominerals [29,30,31]. Moreover, more efforts are being made in order to connect the recovery of metal(loid)s-polluted areas to the valorisation of plants of economic interest [32,33]. Considering the importance of the mining-related environmental problem and the fundamental usefulness of plants in solving it, this review aims to achieve the following: (i) analyse the main issues related to mine sites and mine waste surfaces polluted with metal(loid)s, with a special focus on the Mediterranean Basin; (ii) summarise the main plant-based and multidisciplinary approaches to phytoremediation applied in the Mediterranean context; and (iii) provide a selection of Mediterranean native plant species studied for phytoremediation purposes from a vegetation dynamic perspective. To achieve this, we initially identified the most significant abandoned mining sites (inactive for at least 50 years) through a thorough literature search. We selected these sites on the basis of the quantity and quality of information available regarding historical metal mining activities and the use of indigenous metallophytes. Once the sites were identified, we gathered data through the scientific online databases ScienceDirect, Scopus, and Google Scholar. Compared to other ones, this review has an innovative multidisciplinary approach to analyse the different issues of mining-related problems. Moreover, we chose to organise useful plant species according to their biological forms, vegetational types, and ecology. This last aspect, to the best of our knowledge, is poorly investigated, although it is a crucial perspective to be taken into account for long-term and effective restoration plans, which careful considers the ecology and evolution of natural vegetation specific to each target area.

## 2. Main Mediterranean Ores and Mine Pollution Hotspots

**Figure 1:**
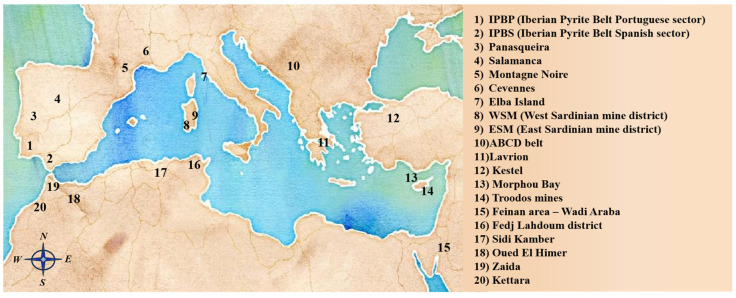
Main metallic mine ‘macro areas’ mentioned throughout the text. Authors’ creation.

The majority of metal mining sites, distributed from the western to the eastern Mediterranean Basin, are related to sulphide ores (Figure 1). Spain and Portugal have had an important mining activity, and the main ores are in the Iberian Pyrite Belt (IPB), one of the biggest sulphide ores of western Europe [34]. São Domingos, Caveira, Rosalgar, Rio Tinto, and Aznalcóllar are some of the most important mining sites in the IPB, some of which are now abandoned with few reclamations [35,36,37]. The area of Aznalcóllar (Seville) is of particular concern as, in 1998, a mine dump crumbled in the Guadiamar river, spilling four million m^3^ of polluted materials rich in metal(loid)s into its course [38,39] and compromising the soils for a surface of 55 km^2^ [40]. An emergency clean-up of the sludge and contaminated topsoil was carried out, even if the underlying soils remained irreparably contaminated. Hence, the regional administration acquired these lands in order to begin a large phytomanagement action using Mediterranean plant species [34].

Not only the IPB mining sites have important effects on humans and ecosystems in Spain and Portugal; also noteworthy are the tungsten (W) mines in the west of the Iberian Peninsula, such as Panasqueira (Central Portugal; [41,42]), and the mining area of Retortillo (Salamanca, Spain), where the most important uranium (U) mine in Europe is located [43]. Spain became a strategic region with regard to W during the First and Second World War, and on a smaller scale, during the Korean War and Spanish Civil War [41]. It should be noted that arsenopyrite, which contains about 30% of As, is rejected with the tailings [44]. Therefore, the potential leaching of As from W waste muds is an evident risk of toxicity for humans and the environment. As far as U is concerned, the International Atomic Energy Agency estimates that 2.4 million m^3^ (25 ha) of U mine waste are left without reclamation in Spain [45]. Due to the high radiological risks, these areas must be restored. For instance, the decommissioning of the Villar de Peralonso mine in Salamanca has not been carried out yet, even though extraction ceased in 1970 [46]. No less important is the dispersion of radioactive dust particles into the atmosphere via the erosion of waste dumps. A regional scale survey carried out in the vicinity of old Spanish U mines estimated a mean annual effective dose ranging from 3.2 to 5.1 mSv per year, which is between 1.2 and 2 times higher than the average national value [47].

In Italy, mine exploitation was carried out mainly in Sardinia and Tuscany. Sardinia is an Italian island where one of Europe’s most important mine poles of the XX century was located. The richest ores (Zn, Pb, and Cd) are in the Sulcis-Iglesiente (SW Sardinia [17,48]) and in the Sarrabus-Gerrei historical regions (SE Sardinia, Pb and As [49,50]). The Sardinian mining industry ceased its activities in 1997, and in most cases, no reclamation actions were undertaken, leaving ca. 70 mm^3^ of abandoned waste materials [51]. Elba Island (Tuscany, Italy) was an important Fe mining area in Italy [52,53]. Ore exploitation ceased in 1981, leaving many abandoned ore mines and waste dumps. Metal and metalloids related to Fe extraction and processing were As, Cr, Co, Cu, Pb, Ni, and Zn [54].

In France, mine exploitation was developed since the II century B.C. [55], mainly in the eastern Pyrenees, the Cevennes, and the Montagne Noire regions, and it was devoted to the extraction of coal and different metals (Fe, Sb, Cu, Pb, Zn, and Al [56,57]). Today, most of these are no longer exploited, but mining waste remains in the surroundings and constitutes potential sources of contamination, with high concentrations of both dissolved and suspended Zn and Pb in downstream rivers [7,58].

The Balkan Peninsula was intensively mined since the Roman and medieval periods, hosting numerous hydrothermal Pb–Zn–Ag–Cu–Au deposits within the Alpine–Balkan–Carpathian–Dinaride (ABCD) belt (Serbo-Macedonian massif, Rhodope Mountains [59]). This belt is one of the world’s oldest mining areas, which played a major role in the history of European civilisations [60].

The main Mediterranean Greek ores, consisting in both base and precious metals, are located in the South Aegean active volcanic and in the Attic–Cycladic ore belt. In particular, Lavrion in southeastern Attica and Lefka-Xeros in Cyprus were some of the main Greek mine areas in the XIX century [7]. Mine exploitation ceased in both areas, and large quantities of polluted materials have been left abandoned with scarce or no remediation actions [61,62].

A large variety of minerals and raw materials have been exploited in Turkey, and several mine sites are present, both inactive and operating, and also of historical interest (i.e., Kestel mines for tin [63]).

In the Near East, Jordan hosts several Cu, Mn, Au, U, and Zr ores; among them, cupreous ones are considered among the oldest in the world [64,65,66]. Copper ores are located in the Feinan area, and the central and southern part of Wadi Araba (South Jordan) and are considered one of the largest sources of copper ore in the Middle East, exploited for a long period (from 6800 B.C. to about AD 1000 [67]).

Along the southern rim of the Mediterranean Basin, Algeria (Sidi-Kamber), Morocco (Zaida mine, Oued El Himer, Kettara mine), and Tunisia (Fedj Lahdoum) had significant deposits of Co, Cu, Fe, Mn, Pb, Sb, and Zn; however, most of them were abandoned after independence, leaving them with few reclamation actions (Figure 1 [7,68,69]).

## 3. Mine Wastes: Main Issues in the Mediterranean Climate Frame and Consequences on Human Health

Mine waste materials, such as mine tailings, derive from the mineral separation process aimed at extracting the metals from the mineral rocks [16,70]. These materials can be of different sizes, but the finest (sands and muds) are the most frequent and harmful ones. Despite their scarce economic value, they are nevertheless enriched in metal(loid)s such as As, Sb, Cd, Cu, Mn, Zn, and Pb up to 50 mg/kg [7,16,70]. Moreover, they are lacking in organic matter (i.e., N, K, and P) nutrients, but are rich in metal salts [36]. In the past, these materials have been generally accumulated in dumps or along the riverbanks, often without control, creating dangerous pollution spots. Hence, the incorrect disposal of mine wastes currently represents an extreme environmental and health risk, and even more so if these materials are abandoned without any kind of control or management. Indeed, this inappropriate arrangement promotes the dispersion of contaminants through atmospheric agents in the waters, soils, and biosphere [36], which is strictly influenced by topography, climate conditions (i.e., water season availability, and the main wind and its exposure), the size of waste materials, and the presence of plant canopy [7,71,72,73,74].

As far as climatic conditions are concerned, the Mediterranean climate is characterised by mild winters and hot and dry summers. These particular characteristics of the Mediterranean climate influence the dispersion of contaminants in a very particular way: indeed, the load of polluted metals in the surrounding environment can vary with season and water availability [6]. Intense and concentrated autumn rainfall can occur, and consequently cause flash floods which mobilise large amounts of polluted materials. In arid and semiarid environments, extreme temperatures (especially on the tailings’ surface), low precipitation, and high winds also contribute to the development of extremely high salt concentrations, ranging up to 22 dS/m, due to high evaporation and low water infiltration [70,75]. Moreover, the dissolved ions in acid mine drainage (AMD) precipitate as sulphates, in a cyclic process of evaporation–dissolution that generates metastable compounds, are easily carried by winds [76]. These sulphates are recognised as “efflorescent salts” that dissolve at the first rain after the dry season, releasing acidic compounds and metals to the environment [77]. AMD can decrease the pH of waters, causing detrimental effects on local flora and fauna. Moreover, it can also enhance the transfer of dissolved metal(loid)s through streams and rivers. This last phenomenon is limited in the Mediterranean biogeographic region because mine ores are often encased or hosted close to carbonate rocks which are able to buffer the pH [7], although several cases of AMD were reported in the literature for Spain in the IPB [78,79], for Sardinia at the Montevecchio-Ingurtosu and Baccu Locci mine sites [6,72], for France, and for Cyprus [80,81]. These adverse conditions can persist for a long time after mine closure and, especially if polluted sites are close to human settlements, confirm the need to properly manage the disposal of abandoned waste materials. In this frame, the possible adverse effect of metal pollution was evaluated by the epidemiological study of Biggeri et al. [1], which showed a higher incidence of malignant lung tumours and respiratory diseases in inhabitants living near mines sites, compared to other parts of Sardinia. Later, the studies of Sanna et al. [2] and Varrica et al. [3] reported higher concentrations of several metal(loid)s in the hair of children living in the Sulcis-Iglesiente area (SW Sardinia) than those living far from mine areas and in unpolluted sites. Accordingly, the Italian law established that the mineralised areas of Sardinia are at high risk of environmental crisis and a possible threat for health (Italian Legislative Decree no. 334/1999). Similar observations were also made on inhabitants living close to the Saõ Domingos mine area (Portugal), who showed higher concentrations of Cd, Cu, and As in their scalp hair than people living several kilometres away [82]. In this last study, the most common exposure pathway seems to be foodstuff like the milk and cheese of cattle grazing in these areas.

## 4. Phytoremediation of Mine Areas in the Mediterranean Biogeographic Region

Although mine wastes are poor in nutrients and organic matter, and enriched in metal(loid)s, several plant species are able to colonise these substrates [17], and even reach high degrees of plant diversity [19,73,74,83] with the presence of some site-specific endemic *taxa*, like *Erica andevalensis* Cabezudo & J. Rivera which grows exclusively in the IPB area on polluted substrates [37,84,85,86,87,88], or *Limonium merxmuelleri* Erben subsp. *merxmuelleri*, which is exclusive to the metalliferous ring of SW Sardinia [73,89]. Several native *taxa* which colonise these polluted substrates have developed different mechanisms to survive in these stressful conditions. Metallophytes are plant species that have developed biological mechanisms to resist to, tolerate, or thrive alongside metal(loid)s, and can also be exclusive to their native metalliferous substrate [90]. Metallophytes can be categorised as absolute, which are found only on metalliferous substrates, and facultative, that can also grow on non-metalliferous ones [91,92]. The use of metallophytes for phytoremediation is largely recognised as a viable practice for the reclamation of mine sites, because it is an eco-friendly, sustainable, and efficient alternative to conventional technologies [36]. Some species can immobilise pollutants into hypogeal organs or the rhizosphere, limiting their dispersion in the surrounding environments and making them suitable for phytostabilisation (i.e., excluder *taxa*), whereas others are able to accumulate metals in high amounts in epigean organs, thus being useful for phytoextraction (i.e., accumulator and hyperaccumulator *taxa*). Among the different phytoremediation technologies, phytostabilisation promotes the ecological integration of mining structures within their surrounding landscape matrix, and supports the maintenance of long-term plant canopy and the reduction of visual impact [36,93]. On the contrary, phytoextraction is oriented to the recovery of metal(loid)s from the substrate [94]. So, *taxa* with a short life cycle, high biomass, and fast growth are desirable [95]. When they end their life cycle, they are harvested in order to avoid that pollutants return to the substrate [96,97,98]. Furthermore, particular attention must be given to phytoextractors to prevent their consumption and entry into the food chain. After removal, plants are disposed as hazardous waste or incinerated for metal recovery, a process called phytomining [16,70,99]. In order to improve the yield of recovered metals and shorten the time of restoration, a seasonal rotation of plants can be considered, using drought-resistant species for summer and cold-resistant ones for winter [100].

Different biological indexes can be calculated in order to evaluate the behaviour of metal-tolerant *taxa*. Some examples are the biological accumulation coefficient (BAC), the biological concentration factor (BCF), and the translocation factor (TF). In detail, BAC (=[Mt] epigean organs/[Mt] substrate) is used to estimate the accumulation of metal(loid)s in epigean organs with respect of the content in the substrate, and it is described as the ratio between the concentrations of the pollutants ([Mt]) in them (Brooks 1998) [101]. BCF (=[Mt] hypogeal organs/[Mt] substrate) allows researchers to evaluate the accumulation ability of hypogeal organs, and is described as the ratio between the concentrations of a certain pollutant ([Mt]) in the hypogeal organs and the substrate [102]. TF (=[Mt] epigean organs/[Mt] hypogeal organs) allows researchers to evaluate the metal(loid) translocation from hypogeal to epigean organs, as the ratio between the concentrations of the pollutant ([Mt]) in these compartments [101]. Table A1 (Appendix A) presents further information concerning BAC, BCF, and TF, as well as information about the uptake of metal(loid)s of several plant *taxa* discussed in the following paragraphs.

In this frame, native plants are adapted to local climate and to stressful substrate conditions, and can colonise waste material surfaces, forming a plant canopy [36,103]. Moreover, several pioneer plant species of these environments can be nurse plants or ecosystem engineers, as detected for *Pinus halepensis* Mill. or *Atriplex halimus* L. in Spain [93]. These *taxa* have a crucial role in favouring the micro-niche formation and allowing the subsequent establishment of other species [93,104]. Indeed, they are able to improve substrate fertility through root exudates and giving litter, reduce solar radiation, and increase water availability [93,105].

**Figure 2:**
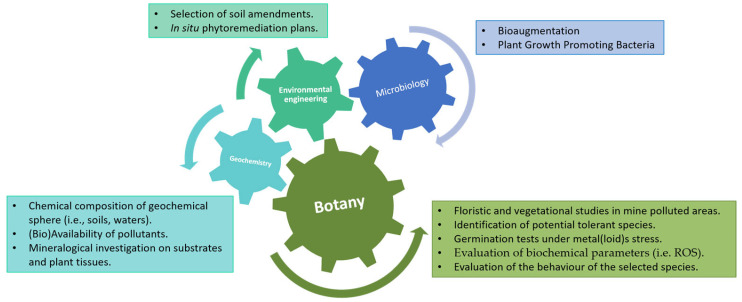
Sketch of the multidisciplinary approach. Authors’ creation.

A deep knowledge concerning native vegetation that spontaneously colonises tailings is fundamental in order to favour the establishment and development of natural vegetation [19,36]. In the light of all the above aspects, a multidisciplinary approach (Figure 2), including geobotany, geochemistry, microbiology, and environmental engineering, is evidently necessary when planning a phytoremediation action. In addition to selecting the most suitable species based on floristic and vegetation studies, the geochemistry of the substrate involved is crucial. The metal(loid)s bioavailability is controlled by soil physico-chemical parameters, such as pH, redox potential, texture, organic matter content, etc., and by biological parameters (plants and microorganisms) [106]. For instance, for the acquisition of Fe, plants acidify the substrates causing the reduction of Fe^3^^+^ to its soluble form Fe^2^^+^, that is transported across the root plasma membrane [107]. The excretion of proton and phenolic compounds by the roots may increase the solubility of Fe ions in the rhizosphere [106,108]. Similarly, understanding the microbial component within the rhizosphere or the germination behaviour in the presence of metal(loid)s is essential. This “multi-tool” approach has been used, for example, for the Sardinian case studies of *Pistacia lentiscus* L. [13,15,16,18,30] and *Helichrysum microphyllum* Cambess. subsp. *tyrrhenicum* Bacch., Brullo & Giusso [17,109,110,111]. A similar approach was applied in the Iberian region for *E. andevalensis* [84,85,86,87,88,112,113,114,115].

In the last 10 years, several studies have promoted complementary research to the canonical investigation about metal accumulation in plant tissues and the ratio between them (i.e., application of biological indexes of accumulation, like BCF). Relatively recent studies concern aspects related to the ecophysiology of germination under metal(loid)s stress [17,110], the evaluation of the antioxidant response of the plant species [53,87,112,116,117,118,119,120], and the presence of biominerals involved in metal mobility [30,31,109,121,122,123]. Whilst the last two topics are quite common in the Mediterranean Basin, the effects of metal(loid)s during seed germination and in the early seedling development of Mediterranean vascular plant species are scarcely investigated. This information, while not directly applicable to the field of phytoremediation/extraction, is, in our opinion, crucial for providing a more comprehensive understanding of the ecophysiology of a *taxon* that can be used for such purposes. Another promising understudied aspect, especially in the Mediterranean Basin, is the role of plant-associated microbes in facilitating metal(loid)s uptake through various biogeochemical processes including translocation, transformation, chelation, immobilisation, solubilisation, precipitation, volatilisation, and complexation [12,124]. For instance, the amendment of the fungus *Simplicillium chinense* F.Liu & L.Cai was proven to enhance the phytoextraction of Cd and Pb by *Phragmites australis* (Cav.) Trin. ex Steud. [125].

## 5. Mediterranean Plant Species for Phytoremediation

The first studies on the use of metallophytes for phytoremediation date to the end of the XIX century, with the first report of a calaminarian species, *Thlaspi caerulescens* J. Presl & C. Presl [126], becoming more consistent during the XX century. In almost 150 years since the first report about the accumulation of metals in a plant, several species have been investigated. Attempts to create a database on metallophytes have been made, like Environment Canada’s PHYTOREM database or the METALS (metal-accumulating plants) database maintained by the Environmental Consultancy of the University of Sheffield (ECUS Ltd., Sheffield, UK). However, a comprehensive Med-scale database is nowadays far, even though an interesting review about metal(loid)-accumulating Italian plants was carried out, but including also non-invasive alien *taxa* [127].

For the Mediterranean biogeographic region, we found studies for 37 *taxa*, belonging to 18 families and 26 genera (Table 1).

Most of the investigated species among metal-tolerant plants and hyperaccumulators comprise a few families, such as Brassicaceae (four *taxa* of four different genera), Cistaceae (six *taxa* of six different genera), and Lamiaceae (six *taxa* of three different genera). In particular, the Brassicaceae are likely showing a high rate of *taxa* with a potential for phytoextraction. These families are among the most representative in the Mediterranean floras (e.g., [19,128]), and include many species that have developed stress-tolerance mechanisms, mostly related to drought and excessive ion concentration [129,130]. However, it is noteworthy that several *taxa* representing different botanical families, such as the Juncaceae, Anacardiaceae, Fabaceae, Asteraceae, Poaceae, and Euphorbiaceae, seem to have received relatively less attention, despite their prevalent presence in mining environments. The limited exploration of these *taxa* can be attributed to the practical challenges associated with their cultivation and experimental implementation, rather than stemming solely from reasons related to their physiology.

**Figure 3:**
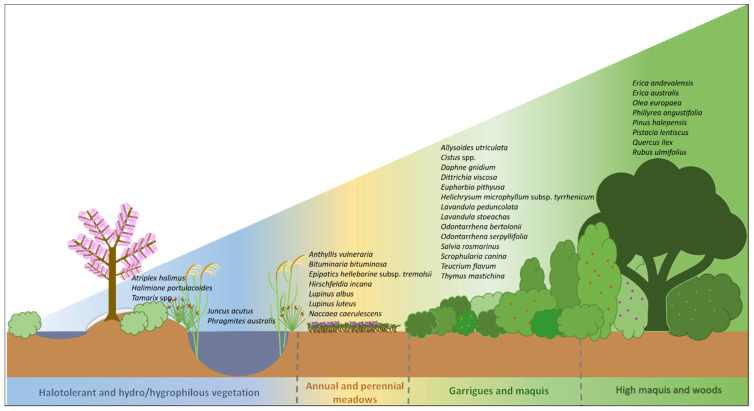
Visual representation of the distribution of *taxa* among the four proposed categories. Authors’ creation.

In consideration of the natural vegetation dynamics, these *taxa* can be attributed to four large categories, considering their biological forms, vegetational types, and ecology: (1) halotolerant and hydro/hygrophilous vegetation; (2) annual and perennial meadows; (3) garrigues and maquis; and (4) high maquis and woods. A schematic representation of these categories is given in Figure 3.

### 5.1. Halotolerant and Hydro/Hygrophilous Vegetation

Halotolerant species are promising candidates not only for the management of ecosystems affected by salt stress, but also for the reclamation of metal(loid)-polluted sites. In this category belong not only halophytes stricto sensu, but also hydro/hygrophilous *taxa* that possess an intrinsic tolerance to salt stress (see [129,131]). It is noteworthy that several Mediterranean mining contexts are rich in terms of different kinds of salts, in particular, sulphides of various metals or sulphate efflorescent salts [77].

*Juncus acutus* L. (Juncaceae) and *Phragmites australis* (Poaceae) are two of the most investigated candidates for wet environments. Both species are halotolerant-hygrophilous species which also grow in the extremely polluted environments of mine areas [16]. Several studies showed that *J. acutus* is able to tolerate a high concentration of metals (Zn and Pb) and metalloids (Sb and As), which are mainly accumulated in its roots, showing its suitability for phytostabilisation (BAC, BCF, and TF < 1 Table A1) [115,132]). Furthermore, recent studies highlighted the presence of different Zn biomineralisation and complexation processes in its tissues, mainly driven by cysteine and citrate compounds [122,123]. This plant species can react to various conditions, tuning the kind of biomineralisation on the basis of the local geochemical and mineralogical conditions. *Phragmites australis* can be used as a phytostabiliser species. Metals like Zn, Pb, and Cd are mainly accumulated into roots with BAC and TF < 0.1 (Table A1 [16,133]). Moreover, X-ray microscopy (STXM) indicates that Fe and Zn are co-located together with Si and Al on the external part of the roots, and partially translocated to the stem and leaves [134]. Likewise observed in another pioneer species of mine environments [109], Zn speciation in *P. australis* also comprises organo-molecules such as Zn cysteine [134], which acts as a detoxing agent.

*Tamarix africana* Poir., *T. gallica* L. (Tamaricaceae), *Halimione portulacoides* (L.) Aellen, and *Atriplex halimus* (Amaranthaceae) are halotolerant species suitable for the ecological restoration of polluted salt marsh soils [26]. *Tamarix africana* and *T. gallica* show an extreme ability to accumulate metal(loid)s, in particular, As, Cd, and Hg (Table A1 [34,121,135,136]). *Tamarix africana* is able to store the contaminants in its roots, with small concentrations of pollutants in its epigean parts [137]. *Tamarix gallica* was tested in hydroponic culture to Hg and As stress, showing a great ability to accumulate Hg and As, and an increase in the production of thiol compounds, which seem to be important in this plant’s resistance to these elements [121]. *Halimione portulacoides* limits the translocation of metal(loid)s in the epigean tissues, and its non-accumulator behaviour indicates a potential candidate for the phytostabilisation of contaminated salt marsh soils (TF < 0.1, Table A1 [136]). *Atriplex halimus* has been used in different contexts for the stabilisation of soils against erosive forces because of its deep root system, but it also showed a high accumulation of Zn and Cd in its shoots (Table A1 [138]).

### 5.2. Annual and Perennial Meadows

Although herbaceous *taxa* present small and low biomass, they can be advantageous for their pioneer and fast-growing behaviour. On the other hand, they easily release metals accumulated in their epigean organs after their death [31]. Several herbaceous species of Brassicaceae and Fabaceae are recognised as metallophytes and metal-accumulating species [139,140,141,142,143]. These *taxa* are able to accumulate and tolerate high quantities of a large number of metal(loid)s [130]. In particular, the first reported cases of Ni and Zn hyperaccumulation in plants were found in Brassicaceae, and about 90 *taxa* of this family in the temperate regions are recognised as Ni hyperaccumulators [144]. As already mentioned, the first studies on heavy metal transport in plants were on *Noccaea caerulescens* (J.Presl & C.Presl) F.K.Mey., nowadays considered a model Zn/Cd hyperaccumulator [145,146]. In subsequent years, numerous tolerant species were discovered such as *Hirschfeldia incana* (L.) Lagr.-Foss. which is a Cu, Tl, and Zn accumulator [147,148,149] and a Pb hyper-accumulator (TF (Pb) > 1, see Table A1 [126]).

Among Fabaceae, some genera like *Anthyllis*, *Bituminaria*, *Lotus*, *Ornithopus*, *Trifolium*, and *Vicia* were found to be tolerant to metal(loid)-pollution [150] and suitable for phytostabilisation, such as *Anthyllis vulneraria* L., *Bituminaria bituminosa* (L.) C.H.Stirt, *Lupinus luteus* L., and *L. albus* L. [53,116,151,152]. These species are adapted to drought and polymetallic and metalloid pollution, tolerating high concentrations of As, Cu, Pb, and Zn (Table A1). Using Fabaceae as a pioneer species has various advantages: they improve soil characteristics in terms of nutrients and organic content, which makes the growth of other plant species possible. Indeed, they have an important role in providing N, increasing the fertility of substrates, and consequently increasing rhizobacteria and arbuscular mycorrhiza diversity which helps to immobilise metals and to enhance the growth of plants [153].

Orchidaceae can also be present in mine environments, such as *Epipactis helleborine* (L.) Crantz subsp. *tremolsii* (Pau) E.Klein, which spontaneously colonised the tailing heap of the Barraxiutta mine site (SW Sardinia), accumulating and translocating metal(loid)s in its organs [154]. The presence of Orchidaceae on contaminated substrates is justified by the presence of mycorrhizal symbiosis with free-living soil fungi that help orchids to protect themselves against the effects of metal(loid)s [154,155].

### 5.3. Garrigues and Maquis

Chamaephytic and nanophanaerophytic shrubs are often candidates for phytoremediation, especially in xeric Mediterranean contexts, where they are part of the mid-successional stages of ruderal and stress-tolerant scrublands, and have also a nurse and engineer function [156]. As in the case of herbaceous plants, the Fabaceae and Brassicaceae families are also representative among hyperaccumulator shrubs [141,142,157,158,159]. One of the first reports in this sense was for the Tuscan endemic *Odontarrhena bertolonii* (Desv.) Jord. & Fourr. [160], and, later, for *Alyssoides utriculata* (L.) Medik. and *Alyssum serpyllifolium* Desf. subsp. *lusitanicum* T.R.Dudley & P.Silva in Portugal, all of which are capable of hyperaccumulating Ni (Table A1 [144,161]). Several Fabaceae, such as shrubs belonging to the *Cytisus*, *Genista*, and *Ononis* genera, were found to be particularly tolerant to metal(loid)s pollution [150]. For instance, *Genista insularis* Bacch., Brullo & Feoli Chiapella subsp. *fodinae* Bacch., Brullo & Feoli Chiapella is an endemic shrub of the Iglesiente subsector (Su Zurfuru mine area, SW Sardinia), colonising metalliferous (Zn and Pb) metamorphic loose substrates [162]. It is emblematic that the subspecific epithet refers to the Latin “fodina”, meaning “mine”. Similarly to the above-mentioned shrubs, some Asteraceae comprise heliophilous and edaphically indifferent plants, growing in a large variety of mine waste surfaces, from coarse to fine substrates, from less stabilised dumps to aged ones. Asteraceae members behave as pioneer species [73], and show a high metal tolerance capability, making them suitable for phytostabilisation. For instance, *H. microphyllum* subsp. *tyrrhenicum* is a good candidate for the phytostabilisation of the Cyrno-Sardinian mine areas (see Table A1 for BAC, BCF, and TF values [17,109,110,163,164]). Moreover, this *taxon* is the topic of a multidisciplinary case study, as it was subjected to germination tests to assess the ecophysiology of seed germination under different Zn, Pb, and As concentrations, and to a mineralogical investigation of the soil plant system [109,110,111]. These studies showed that this *taxon* has (i) a good germination capability even when subjected to high concentrations of Zn, Pb, and As; (ii) a much higher toxicity of arsenite (As III) than arsenate (As V) in terms of seed germination, cotyledon emergence, and mortality of young seedlings; and (iii) the presence of a self-built exclusion mechanism that is expressed through the production of root/leaf biominerals and of Zn organic complexes which act as detoxing agents (i.e., Zn cysteine and Zn malate). Although Asteraceae are generally considered metal excluders and only suitable for phytostabilisation, further species were found to be effective for phytoextraction, such as *Dittrichia viscosa* (L.) Greuter [51,117,165,166,167], which accumulates high concentrations of As, Cd, Pb, and Zn (Table A1). Later studies showed no damage on the metabolic system and no significant alteration in the pigment and foliar metabolites, or in the antioxidant enzymatic activity, if compared with specimen growth in an unpolluted site [53].

Several Lamiaceae are heliophilous shrubby woody species commonly found in Mediterranean scrubs and garrigues, and are able to tolerate heat and drought, growing in poor, dry, sandy, and rocky soils. Lamiaceae are generally not considered as metallophytes, but some studies showed their presence in metal(loid)-polluted areas or on geochemically anomalous ones. The members of this family are rarely found on highly polluted mine waste dumps of fine granulometry; when present on mine sites, they colonise low polluted and more coarse and mixed debris, especially on aged dumps where pollutants were washed out by weathering. Several studies highlighted their potential in phytostabilisation actions. For instance, *Lavandula stoechas* L. subsp. *stoechas* was found to be able to accumulate Mn and Co from serpentinised rocks [168] and to translocate Cu, Pb, and Zn from its roots to its epigean organs (TF > 1, Table A1; [53]). *Salvia rosmarinus* Spenn. can accumulate Zn, Pb, and Cd into its roots, limiting the translocation into its epigean parts (BAC < 1, Table A1 [132,133,169]). *Teucrium flavum* L. subsp. *glaucum* (Jord. & Fourr.) Ronniger is able to accumulate high concentrations of Zn and Pb in its epigean organs [170]. As far as Iberian endemic Lamiaceae are concerned, some *taxa* can be interesting for the phytostabilisation of specific mine areas of the Iberian Peninsula, like *Lavandula stoechas* subsp. *luisieri* (Roizera) Roizera, *L. pedunculata* (Mill.) Cav., and *Thymus mastichina* (L.) L. In particular, *L. stoechas* subsp. *luisieri* and *L. pedunculata* are tolerant species toward several metal(loid)s with exclusion behaviour (see Table A1 [118,171]), whereas *T. mastichina* highly accumulates Ni, Cr, Co, Mn, Zn, and As in its aboveground parts (TF > 1, see Table A1 [172,173]). Some Lamiaceae can be pioneer entities with a preparatory feature for the potential establishment of a more evolved vegetation, whereas others can be found together with Cistaceae in most developed vegetation communities.

Cistaceae are widespread all over the Mediterranean Basin, including heliophylous shrubby species with an acidophilous and calcifuge behaviour (with some exceptions like *Cistus albidus* L.). Most efforts were focused on *Cistus* species, showing their potential use for phytostabilisation considering their tolerance towards As, Cu, Fe, Pb, and Zn, such as *C. albidus*, *C. ladanifer* L., *C. populifolius* L., and *C. salviifolius* L. (see Table A1 for more details [51,53,115,118,119,133,137,165,166,174,175,176,177,178,179]). Moreover, *C. ladanifer* and *C. populifolius* can present inter- and intra-population variations in terms of the accumulation and translocation of chemical elements [84,133,180], and their behaviour may depend on specific mine environments (Table A1). Among *Cistus* species, *C. libanotis* L. was found to be suitable for the phytoextraction of Pb and Cd [179,181], showing an accumulator behaviour for Pb and hyperaccumulator behaviour for Cd. Moreover, *C. monspeliensis* L. showed an accumulator behaviour for Zn, Mn, and Cd, whereas it can be considered tolerant and an excluder for As, Cu, Ni, and Sb [35,178]. This extreme adaptation can be explained through a mechanism to minimise the harmful effects of the oxidative stress caused by high levels of As [119].

Among Scrophulariaceae, *Scrophularia canina* L. is a chamaephytic, heliophilous, and thermophilous plant described as tolerant and suitable for the phytostabilisation of the Fedj Lahdoum and Monteponi mine areas, in Tunisia and Sardinia, respectively [73,132]. The putative subspecies, *S. canina* L. subsp. *bicolor*, is endemic to Sardinia, Corsica, and Sicily. It colonises generally pebbly substrates and grows on gravel both natural and deriving from mine activities, taking part in pioneer plant assemblages of mine environments [73]. An *in situ* phytoremediation experiment carried out on mine wastes of SW Sardinia [170] showed that this plant species is able to accumulate high concentrations in its aerial tissues. Recent studies demonstrated its suitability for phytoextraction with a translocation factor (TF) > 1 for both Zn and Pb (Table A1 [15,182]).

*Daphne gnidium* L. (Thymelaeaceae) is another nanophanaerophyte species common to Mediterranean maquis, growing on rocky and degraded soils. It is also able to grow in extreme environmental conditions like the Saõ Domingos mine (Portugal). It was found to be able to tolerate high concentrations of metal(loid)s such as As, Sb, and Zn and can be considered as a potential phytostabiliser species for the sulphide mining areas of IPB and other Mediterranean mine sites [183], as was also observed in the Fedj Lahdoum mine area in Tunisia (BAC and TF < 1, Table A1 [132]).

*Euphorbia pithyusa* L. subsp. *cupanii* (Guss. ex Bertol.) Radcl.–Sm. (Euphorbiaceae), an heliophilous, edaphically indifferent, and endemic species of Sardinia, Sicily, and Corsica, is a pioneer *taxon* of mine environments, and several studies carried out on highly polluted mine areas of Sardinia showed its application in phytostabilisation. Indeed, it can accumulate up to 300 mg/kg Zn in its leaves [166], and is able to maintain constant Zn and Pb concentrations in its leaves independently of the respective soil concentrations [51]. This aspect shows that the presence of such an adaptation mechanism is able to limit the accumulation of metal(loid)s in aerial organs. This behaviour was subsequently confirmed by the study of Medas et al. [31], in which this species’ capacity of building a self-tolerance mechanism against high Zn concentrations, through Zn biomineral formation in its root tissues, was revealed.

### 5.4. High Maquis and Woods

Considering their extremely long life cycle, the plant species belonging to this category can be considered for long-term environmental rehabilitation.

*Pistacia lentiscus* L. (Anacardiaceae) is probably the most used phytostabiliser species in the Mediterranean (BCF, BAC and TF < 1, Table A1 [15,16,18,34,133,182]). It is able to tolerate extremely high concentrations of As, Cd, Pb, and Zn, building its own tolerance mechanism through the formation of specific biominerals in the surface of its roots [30]. Furthermore, synergistic interactions between *P. lentiscus* and several endophytic bacterial strains were observed, showing that the survival and growth of this plant species is improved [13,14].

Ericaceae are also known for being capable of absorbing essential macro- and micronutrients and sequestering excess toxic elements in their root bark and rhizosphere soil [37,84,86,171], as well as having an intrinsic ability to tolerate high levels of Al and Mn [86]. The case of *Erica andevalensis* is of particular interest for phytoremediation in addition to the ecological implications. This *taxon* is an endemic of the SW Iberian Peninsula, described for the first time in Spain by Cabezudo and Rivera [184]. It grows specifically in the acidic and metal-enriched soils of the IPB [112,114], and is classified as an endangered species by the Andalusian Regional Government [86]. This plant grows on the banks of rivers affected by AMD as a monospecific population or together with *Erica australis* L., in combination of other shrubs such as *C. ladanifer* [85,86,88,112], or in coarse and very dry mine tailings [184]. *Erica andevalensis* behaves as an Mn-accumulator and Al-tolerant species [84,112]. Moreover, it can tolerate very high concentrations of As, Pb, and Sb [84,88,115], and it is a Zn excluder [112,114]. The presence of a complex rhizosphere microbiological community, including bacterial clones sharing genes resistant to Ni [112,113], and Fe- and Al-accumulating fungi in its roots [185], can explain the ability of this *taxon* to survive in extreme conditions. Márquez-García and Córdoba [112] showed how antioxidative mechanisms act in *E. andevalensis*: this species counteracts the formation of reactive oxygen species (ROS) by oxidising ascorbic acid, as low levels of ROS do not damage cell structures or physiology without the activation of defensive enzymes.

No less significant for phytoremediation purposes is *E. australis*, a tall evergreen shrub native to the Iberian Peninsula and Morocco, living frequently in disturbed habitats; it can be considered a good candidate for the phytostabilisation and colonisation of soils affected by sulphide mining activities, being Al- and As-tolerant and a Mn-accumulator [37,84,88,112]. *Erica australis* is able to grow in metal(loid)-polluted substrates such as in the IPB region, being a primary coloniser of the Rio Tinto region (Spain), which is characterised by extreme water pH values.

*Rubus ulmifolius* Schott (Rosaceae), a common shrub of the Mediterranean area, was discovered to be a good candidate for the phytoremediation of As-, Ni-, and Pb-polluted substrates [186]. It is able to accumulate these metal(loid)s within its roots, limiting their translocation to its aboveground organs. This characteristic makes the species an excluder *taxon*, hence appropriate for phytostabilisation (BCF < 1, see Table A1). However, the capacity of accumulating Pb and Zn in its leaves and blackberries was found for a species of the same genus, *R*. *fruticosus* L., in Germany [187].

Considering wood *taxa*, *Pinus halepensis* (Pinaceae) is a widespread Mediterranean evergreen tree growing in neutral or slightly alkaline and low fertile soil. It is also able to grow on multi-metal(loid)-polluted mine sites [188,189,190,191,192,193]. In the studies of Kharazian et al. [188,189], *P. halepensis* behaves as an excluder species, tolerating high Zn, Pb, and Cd concentrations and restricting their presence in its hypogeal tissues (BCF and TF < 1, see Table A1).

Among Oleaceae and Fagaceae, some *taxa* were tested for the rehabilitation of mine-polluted areas, like *Quercus ilex* L. subsp. *ballota* (Desf.) Samp., *Olea europaea* L. var. *sylvestris* Brot., and *Phillyrea angustifolia* L., all used for rehabilitation after the accident at the Aznalcóllar mine dump [112,114].

## 6. Discussion and Perspectives for the Future

### 6.1. The Role of Native and Endemic Flora of Mine Areas

Native and endemic flora are already adapted to local climate/edaphic conditions and are part of the local biodiversity heritage. Moreover, they can passively restore, along with time, the plant canopy and mitigate the pollution and dispersal of contaminants in the soil, water, and biosphere. Considering the reclamation problems of mine sites, which are often adjacent to urban areas, policy guidelines and land management should pay greater attention to the use of native/endemic Mediterranean plants with phytoremediation characteristics (metal-tolerant and hyperaccumulators). Among them, the ability of halophytes to survive in saline environments makes these species able to survive in locations with high metal contamination and close to “efflorescent salts” that are a pool of metal(loid)s [70,77]. Some of them, such as the halophilous *H. portulacoides*, have already been investigated, but further native halophytes have a potential for phytoextraction and biomonitoring [194]. The plant communities established on mine waste dumps and tailing dumps are often rich in endemic plant species, due to the peculiar and extreme environmental conditions that favour hyperspecialised and poorly competitive species [195,196,197]. For instance, in the Sardinian mine environmental context, several endemic *taxa*, like *Echium anchusoides* Bacch., Brullo & Selvi, *Genista insularis* subsp. *fodinae*, *Iberis integerrima* Moris, *Limonium merxmuelleri* subsp. *merxmuelleri*, *Linum mulleri* Moris, *Ptilostemon casabonae* (L.) Greuter, *Santolina corsica* Jord. & Fourr., and *Verbascum plantagineum* Moris, are some of the potential metallophytes to be investigated. In our opinion, native and endemic flora of mine areas can also make a contribution to improve human health in the area subjected to mine impact, both from a physical (reducing illness caused by metals like metal poisoning) and from a psychological point of view.

### 6.2. Local Policies and Guidelines for Environmental Restoration

Native and endemic flora of mining areas need more attention in local policies and guidelines for environmental restoration. Several studies about the positive effect of urban green have been carried out [198,199,200], showing the beneficial effect on the physical health and well-being of all cultural and social groups. In the near future, cities will host an increasing number of inhabitants who will have to live in environmental conditions suitable for well-being and quality of life. The objectives of the European Biodiversity Strategy 2030 are being implemented in the member states through green planning and management measures (European Green Deal; Green revolution and ecological transition) to promote tree planting that supports biodiversity, climate mitigation, and resilience [10]. Their ambitious goal is the creation of urban, peri-urban, and extra-urban forests, with the planting of millions of trees in cities, to address air pollution problems, the impact of climate change, and the loss of biodiversity, and improve the quality of life and well-being of citizens [11].

Mining sites can be included as protected areas according to categories defined by IUCN as ‘Protected terrestrial/marine landscapes: terrestrial and/or marine, a protected area where the interaction of people and nature over time has produced an area of distinct character with significant ecological, biological, cultural, and scenic value and where safeguarding the integrity of this interaction is vital to protecting and sustaining the area and its associated nature conservation and other values’ [201,202,203]. Accordingly, some of the sites mentioned here can be—or are already included—in the UNESCO world geosite/geopark network, as is the case of Lavrion in Greece (within the Lavreotiki UNESCO Global Geopark [204]) or the proposed geopark of Sardinia [202]. Moreover, Fois et al. [205] proposed an improvement to Annex I of Directive 92/43/EEC with the new habitat ‘Calaminarian vegetation of mining dumps, tailing dams and quarries’. These are some of the various options for implementing different policies aimed at the integrated protection, valorisation, and sustainable use of these historical and environmental legacies.

### 6.3. The Importance of the Multidisciplinary Approach and Its Future Implementation

As in other bioclimates, a multidisciplinary approach should be preferred in the Mediterranean. Seed germination tests under metal(loid)s and mineralogical investigation together with microbiological survey are some of the approaches to be considered when filling the “tool-box”. Improvements in seed germination studies can be made using a mixture of metal(loid)s or using the waste leachate, which, as far as we are aware, is little used today. A mixture of metal(loid)s allows to mimic ex situ the in situ field concentrations, whereas the waste leachate allows researchers to test the germination behaviour of seeds on soluble fraction of metalloids from the original substrate. In this frame, germplasm banks have another crucial role in storing and testing seeds, both for conservation and as a source of future seedling production for rehabilitation purposes. Nowadays, few germplasm banks in the Mediterranean are strictly dedicated to metallophytes, but several are storing and preserving a great number of metallophyte species [90,206].

The “tool-box” can also be filled by the knowledge of the biochemical response of native plants to metal(loid)s exposure, using different biochemical parameters to assess the health condition of plants. These parameters can be, for instance, chlorophyll production, anthocyanin and glutathione concentrations, as well as malondialdehyde levels. It is a well-known fact that metal(loid)s can negatively interfere in photosynthetic activity and are strictly linked to the production of ROS [207]. Hence, the levels of malondialdehyde, the final product of lipid peroxidation, can be used as an indicator of oxidative stress. On the other hand, the concentration of glutathione, an antioxidant involved in cellular defence against metal(loid)s [207], can give information about the response of plants to metal(loid) exposure. These parameters are, for example, scarcely investigated in metallophyte *taxa* of the Mediterranean Basin, and little is known concerning the antioxidant system of metallophytes, because most of the studies are carried out on “model plants” or under controlled conditions at the laboratory scale [112].

From a phytoremediation perspective, substrate amendments can be used, because they can modify the physical-chemical properties of mine substrates and the bioavailability of metals [17,208,209]. Among them, compost acts by lowering metal availability in substrates, decreases the plant’s tissue uptake, and at the same time increases nutrients and organic matter in substrates [16]. Furthermore, compost improves the texture of the substrate, water retention, and ventilation [17]. Other organic amendments can be promoted, like woodchips, biochar, winery waste, and mushroom residues, giving a second life to organic fractions of municipal solid waste and special waste [21,22,23,24,25].

### 6.4. Phytostabilisation or Phytoextraction?

In our opinion, phytostabilisation must be preferred, and phytoextraction used only for the recovery of rare metals and if the yield is economically advantageous, as was argued in Boi et al. [109]. Indeed, phytoextraction without a detailed recovery plan poses several issues that need to be considered. First, after harvesting, plants must be considered as hazardous waste. Hence, they cannot be used as compost or landfill, and must be managed for the recovery of metals. Incineration produces carbon emissions and air pollution that must be mitigated with engineering solutions. Despite having some potential, it is noteworthy that several authors consider phytoextraction as generally infeasible because of the excessive time required [94,210,211,212], with a time horizon of a human generation for Zn and more than 1000 years for Pb [94]. Last but not least, phytoextraction (and phytomining) are extensive and generally monoculture practices, that can negatively influence the distribution and natural niches of spontaneous *taxa*, thus being a risk for local plant biodiversity [210]. If a phytoextraction strategy is to be pursued, the unique climate of the Mediterranean must also be considered, taking advantage of the seasonal rotations of plants between winter and summer, and selecting species resistant to drought/cold. The Mediterranean climate’s unique climatic conditions and associated pollutant dispersion patterns (as discussed in Section 3) introduce additional constraints that must be taken into account when planning phytoextraction practices.

In conclusion, the utilization of Mediterranean native and endemic plants in the phytoremediation of mining sites should hold a primary position in local policies and environmental restoration guidelines. This point is pivotal and has a cascading effect on the subsequent aspects. The remediation of these areas not only contributes to the preservation of local biodiversity but can also have a positive impact on the well-being of local communities. In this frame, a multidisciplinary approach and a “tool-box” filled with continuously updated methods can effective help the development of phytoremediation activities tailored to local conditions. In the Mediterranean mining context, phytostabilisation is generally recommended, whereas phytoextraction must be considered in few specific cases for phytomining.

## Figures and Tables

**Table 1:** The 18 families and 26 genera represented by the 37 *taxa* (species and subspecies level) analysed in this manuscript and reported in Appendix A. The number of *taxa* per family with potential for phytoextraction is reported in brackets.

Family	N. Genera	N. *taxa*
Amaranthaceae	2	2 (0)
Anacardiaceae	1	1 (0)
Asteraceae	2	2 (1)
Brassicaceae	4	4 (3)
Cistaceae	1	6 (3)
Ericaceae	1	2 (1)
Euphorbiaceae	1	1 (0)
Fabaceae	1	1 (0)
Fagaceae	1	1 (0)
Juncaceae	1	1 (0)
Lamiaceae	3	6 (3)
Oleaceae	2	2 (0)
Pinaceae	1	1 (0)
Poaceae	1	1 (0)
Rosaceae	1	1 (0)
Scrophulariaceae	1	2 (1)
Tamaricaceae	1	2 (1)
Thymelaeaceae	1	1 (0)

## Data Availability

Not applicable.

## Appendix A

**Table A1:** A selection of Mediterranean native plant species studied for phytoremediation of mine sites; BCF = biological concentration factor; BAC = biological accumulation coefficient; TF = translocation factor; R = roots; S = stems L = leaves; EO = epigean organs; *bf* = bioavailable fraction. Missing values indicate unavailable information. *Taxa* reported with asterisks (*) are those showing potential for phytoextraction according to BAC and TF > 1.

Plant Species	Metal(Loid)s	Metal(Loid)s in Plant	BCF	BAC	TF	References
*Alyssoides utriculata* *	Ni	5000 mg/kg R >1000 mg/kg L	-	>1	>1	[133]
*Alyssum serpyllifolium* *	Ni	3405 mg/kg (entire plant)		2.98	4.35	[161]
*Atriplex halimus*	As	6–8 mg/kg L	-	-	-	[138]
	Pb	8–12 mg/kg L	-	-	-	
	Zn	6 mg/kg L	-	-	-	
*Bituminaria bituminosa*	Pb	4.7 mg/kg R 16.7 mg/kg S 4 mg/kg L	0.27	-	0.85	[53]
	Zn	62.9 mg/kg R 107 mg/kg S 70 mg/kg L	0.96	-	1.10	
*Cistus albidus*	As	10.6 mg/kg EO	-	0.13	-	[133]
	Mn	115 mg/kg EO	-	0.09	-	
	Pb	6.74 mg/kg EO	-	0.03	-	
	Zn	124 mg/kg EO	-	0.86	-	
*C. ladanifer* *	Pb	5–69 mg/kg R 5–19 mg/kg S 5–69 mg/kg L	-	1.03	3.87	[176]
	Zn	29–203 mg/kg R 57–214 mg/kg S 124–703 mg/kg L	-	0.05	3.61	
	As	12 mg/kg R 1.7 mg/kg L	-	<1	0.7–0.14	[137]
	Pb	53.88 mg/kg R 55.79 mg/kg L	-	<1	0.67–1.46	
	Zn	41 mg/kg R 147 mg/kg L	-	>1	0.53–4.82	
*C. libanotis*	Pb	823–1820 mg/kg EO	-	-	-	[181]
	Zn	89–1297 mg/kg EO	-	-	-	
*C. monspeliensis* *	As	11.4 mg/kg R 29.7 mg/kg S	-	0.02	2.13	[35]
	Mn	764 mg/kg R 1165 mg/kg EO		3.29	2.18	
	Pb	35 mg/kg R 15.6 mg/kg S	-	0.01	0.60	
	Zn	91 mg/kg R 217 mg/kg S	-	1.60	2.61	
*C. populifolius*	As	0.8–4.8 mg/kg R 0.5–1.1 mg/kg EO	-	<1	0.2–0.7	[175]
	Sb	0.01–0.2 mg/kg R 0.03–0.07 mg/kg EO	-	<1	0.1–0.8	
	Pb	8.9–333 mg/kg R 0.7–20.3 mg/kg EO	-	<1	0.1–0.4	
	As	5.40 mg/kg EO	-	0.053	-	[133]
	Mn	747 mg/kg EO		12	-	
	Pb	5.51 mg/kg EO	-	0.036	-	
	Zn	154 mg/kg EO	-	3.27	-	
*C. salviifolius* *	Pb	185 mg/kg EO	-	-	-	[170]
		1560 mg/kg EO	-	-	-	
	Pb	1.7 mg/kg R 2.1 mg/kg S 3.1 mg/kg L	0.21	-	1.82	[53]
	Zn	26 mg/kg R 58.6 mg/kg S 113.4 mg/kg L	1.55	-	4.36	
	As	0.6–7.2 mg/kg R 0.1–10 mg/kg EO	-	<1	0.2–0.7	[175]
	Sb	0.01–0.9 mg/kg R 0.02–0.2 mg/kg EO	-	<1	0.1–0.8	
	Pb	23.9–312 mg/kg R 1.3–39.6 mg/kg EO	-	<1	0.1–0.4	
	As	5.74 mg/kg EO	-	0.02	-	[133]
	Pb	8.03 mg/kg EO	-	0.04	-	
	Zn	286 mg/kg EO	-	2.74	-	
*Daphne gnidium*	As	4.35 mg/kg EO	-	-	-	[183]
	Zn	104 mg/kg EO	-	-	-	
	Pb	11.5 mg/kg R 2.9 mg/kg S	-	0.002	0.25	[132]
	Zn	243.7 mg/kg R 77.4 mg/kg S	-	0.04	0.32	
*Dittrichia viscosa* subsp. *viscosa **	Zn	2000 mg/kg L	-	-	-	[166]
	Pb	250 mg/kg L	-	-	-	
	As	12.7 mg/kg R 18.5 mg/kg S 26.2 mg/kg L	12.48	-	2.06	[53]
	Pb	4.5 mg/kg R 7.3 mg/kg S 25 mg/kg L	1.69	-	5.56	
	Zn	20.3 mg/kg R 27.4 mg/kg S 133.2 mg/kg L	1.82	-	6.56	
	As	23 mg/kg EO				[167]
	Pb	270 mg/kg EO				
	Zn	640 mg/kg EO				
*Erica andevalensis*	As	2.5–4.4 mg/kg L	-	0.007–0.02	-	[112]
	Mn	790–1100 mg/kg	-	0.8–15	-	
	Pb	2.6–6.31 mg/kg L	-	0.01–0.03	-	
	Zn	15.64–146 mg/kg L	-	0.1–0.3	-	
	As	7.5 mg/kg R 7.5 mg/kg EO	-	0.4 *^bf^*	1.4	[88]
	Mn	500 mg/kg R 1400 mg/kg EO	-	173 *^bf^*	3.1	
	Pb	100 mg/kg R 100 mg/kg EO	-	1 *^bf^*	0.9	
	Zn	60 mg/kg R 40 mg/kg EO	-	3.1 *^bf^*	0.8	
*E. australis **	Mn	500 mg/kg R 325 mg/kg L	-	>1	>1	[37]
	Pb	5 mg/kg R 3 mg/kg L	-	<1	>1	
	Zn	5 mg/kg R 22.5 mg/kg L	-	<1	<1	
	As	26 mg/kg R 3.7 mg/kg EO	-	0.42 *^bf^*	1.08	[88]
	Mn	300 mg/kg R 250 mg/kg EO	-	189 *^bf^*	1.1	
	Pb	125 mg/kg R	-	0.76 *^bf^*	1.2	
	Zn	7.5 mg/kg R 17.5 mg/kg EO	-	8.5 *^bf^*	2.4	
*Euphorbia pithyusa* subsp. *cupanii*	Zn	300 mg/kg L	-	-	-	[170]
*Halimione portulacoides*	As	9.8 mg/kg R 0.6 mg/kg S			0.04–0.1	[136]
	Pb	312.5 mg/kg R 6.9 mg/kg S			0.02–0.03	
	Zn	2.1 mg/kg R 67.9 mg/kg S			0.03–0.04	
*Helichrysum microphyllum* subsp. *tyrrhenicum*	Zn	2600 mg/kg R 3400 mg/kg EO	0.1	0.12	1.7	[110,164]
	Pb	500 mg/kg R 1000 mg/kg EO	0.1	0.20	2.45	
	Cd	20 mg/kg R 19 mg/kg EO	0.2	0.16	1.1	
*Hirschfeldia incana **	Pb	11.7–204.4 mg/kg R 25.4–222.6 mg/kg EO	-	0.009–0.036	1.09–2.16	[132]
	Cd	33.5 mg/kg R		0.07	2.43	
		81.2 mg/kg EO				
	Zn	46.9–528.9 mg/kg R 144.1–911.3 mg/kg EO	-	0.09–0.21	1.72–3.07	
*Juncus acutus*	As	0.0094 mg/kg external R 0.0046 mg/kg inner R 0.00013 mg/kg S	-	-	-	[16]
	Pb	2100 mg/kg external R 580 mg/kg inner R 15 mg/kg S	0.09	0.002	0.03	
	Zn	16,000 mg/kg external R 3500 mg/kg inner R 380 mg/kg S	0.04	0.005	0.1	
	Pb	30 mg/kg R 2.2 mg/kg S	-	0.001	0.07	[132]
	Zn	142.3 mg/kg R 25.8 mg/kg S	-	0.01	0.18	
	As	752 mg/kg R 3.6 mg/kg EO 4 mg/kg F	-	-	-	[115]
	Zn	234 mg/kg R 19 mg/kg EO 38 mg/kg L	2.25	-	-	
*Lavandula stoechas* *	Pb	1.2 mg/kg R 2 mg/kg S 4.6 mg/kg L	0.31	-	3.83	[53]
	Zn	25.8 mg/kg R 29.1 mg/kg S 82.7 mg/kg L	1.13	-	3.21	
*L. stoechas* subsp. *luisieiri* *	Mn	577 mg/kg R 890 mg/kg S	-	<1	>1	[171]
	Zn	47 mg/kg R 131 mg/kg S	-	>1	>1	
	As	1.7 mg/kg EO	-	-	-	[176]
	Pb	6.96 mg/kg EO	-	-	-	
	Zn	139 mg/kg EO	-	-	-	
*L. pedunculata*	As	12.3 mg/kg R 14.6 mg/kg S		<1	>1	[213]
	Pb	26.3 mg/kg R 21.7 mg/kg S		<1	<1	
	Zn	106 mg/kg R 229 mg/kg S		<1	-	
*Noccaea caerulescens*	Zn	53,450 mg/kg L	-	-	-	[143]
	Cd	2908 mg/kg L	-	-	-	
*Olea europaea* var. *sylvestris*	As	0.32 mg/kg L	-	<1	-	[34]
	Cd	0.07 mg/kg L	-	<1	-	
	Pb	0.89 mg/kg L	-	<1	-	
	Zn	42.2 mg/kg L	-	<1	-	
*Phillyrea angustifolia*	As	3.28 mg/kg EO	-	0.06	-	[133]
	Pb	3.94 mg/kg EO	-	0.05	-	
	Zn	73.1 mg/kg EO	-	0.7	-	
*Phragmites australis*	Cd	55 mg/kg R 19 mg/kg EO	-	-	< 0.1	[16]
	Pb	3000 mg/kg R 750 mg/kg EO	3	-	< 0.1	
	Zn	10,000 mg/kg R 4000 mg/kg EO	-	-	< 0.1	
	As	5.18 mg/kg EO	-	0.0014	-	[133]
	Pb	1.17 mg/kg EO	-	0.0001	-	
	Zn	43.1 mg/kg EO	-	0.03	-	
*Pinus halepensis*	Zn	664.65–2710 mg/kg R	0.18	-	0.03–0.19	[189]
	Pb	58.39–735.88 mg/kg R	0.17	-	0.03–0.32	
	Cd	4.86–11.02 mg/kg R	0.19	-	0.04–0.14	
*Pistacia lentiscus*	Zn	450 mg/kg R 350 mg/kg S 150 mg/kg L	<1	<1	<1	[18]
	Pb	30 mg/kg R 25 mg/kg S 10 mg/kg L	<1	<1	<1	
	Hg	0.09 mg/kg R 0.04 mg/kg S 0.250 mg/kg L	<1	<1	2	
	Pb	9.93 mg/kg EO	-	0.13	-	[133]
	Zn	23 mg/kg EO	-	0.25	-	
*Quercus ilex*	As	0.56 mg/kg L	-	<0.2	-	[34]
	Cd	0.21 mg/kg L	-	<0.5	-	
	Pb	2.48 mg/kg L	-	<0.2	-	
	Zn	80 mg/kg L	-	<0.5	-	
*Rubus ulmifolius*	As	<1000 mg/kg R, S, L	0.02–0.16			[186]
	Ni	<1000 mg/kg R				
	Pb	<1000 mg/kg R, S, L	0.08–0.13			
*Salvia rosmarinus*	As	0.79 mg/kg L	-	<1	-	[34]
	Cd	0.04 mg/kg L	-	<1	-	
	Pb	2.01 mg/kg L	-	<1	-	
	Zn	51.2 mg/kg L	-	<1	-	
	As	8.54 mg/kg EO	-	0.01	-	[133]
	Pb	4.89 mg/kg EO	-	0.002	-	
	Zn	95 mg/kg EO	-	0.3	-	
	Cd	1.7 mg/kg R 0.3 mg/kg S	-	0.12	0.18	[132]
	Pb	9.6 mg/kg R 3.2 mg/kg S	-	0.001	0.33	
	Zn	30 mg/kg R 19.5 mg/kg S	-	0.01	0.65	
*Scrophularia canina* subsp. *bicolor **	Pb	120 mg/kg EO	-	-	>1	[15,170]
	Zn	1200 mg/kg EO	-	-	>1	
*S. canina*	Pb	3.8 mg/kg R 5.4 mg/kg S	-	0.001	1.44	[132]
	Zn	20.5 mg/kg R 24.1 mg/kg S	-	0.01	1.17	
*Tamarix africana* *	As	0.10 mg/kg R 0.007 mg/kg S	-	-	0.8	[136]
	Pb	0.6 mg/kg R 0.7 mg/kg S	-	-	1.1	
	Zn	27.8 mg/kg R 56.6 mg/kg S	-	-	2	
*T. gallica*	As	520 mg/kg R 68 mg/kg S	-	-	-	[121]
	Hg	1536 mg/kg R 22 mg/kg S	-	-	-	
*Teucrium flavum* subsp. *glaucum*	Pb	346 mg/kg EO	-	-	-	[170]
	Zn	1130 mg/kg EO	-	-	-	
*Thymus mastichina* *	Zn	4.0–240 mg/kg R 8.0–65.0 mg/kg S 17.5–145 mg/kg L	-	-	1.7–5.8	[173]
	Ni	2.5–100 mg/kg R 2.5–35.0 mg/kg S 4.0–180 mg/kg L	-	-	1.4–3.0	
